# Supplementary material for: Complexome profiling of the Chlamydomonas psb28 mutant reveals TEF5 as an early PSII assembly factor
Source: Plant Cell. 2025 Mar 17;37(6):koaf055. doi: 10.1093/plcell/koaf055 (PMC12164590; doi:10.1093/plcell/koaf055)
Supplement: koaf055_Supplementary_Data [file koaf055_supplementary_data.zip › psb28_interactive.zip!/plots/intensity/qualityPlots/peptides/ASA3_Cre07.g338050.t1.2.html]

### Cre07.g338050.t1.2

Trivial name: ASA3  
SeqCoverage [median]: 44.30  
Mass: 39.73 kDa  
  
 

| Sequence | MissedCleavage | Mass [Da] | wtIntensity | muIntensity | Score |
| --- | --- | --- | --- | --- | --- |
| AIISESIVK | 0 | 958.57 | 58066.41 | 116137.62 | 151.04 |
| AVAGAATSSTIR | 0 | 1103.59 | 84292.19 | 115339.31 | 195.36 |
| AVAGAATSSTIRDLVTSFAEK | 1 | 2094.10 | 1673.85 | 0.00 | 104.88 |
| DLVTSFAEK | 0 | 1008.51 | 65446.92 | 160951.59 | 163.15 |
| FAEAVKPK | 1 | 888.51 | 20175.66 | 32621.98 | 143.12 |
| GDLFVALAR | 0 | 960.54 | 33498.85 | 0.00 | 109.86 |
| GFSELSLAK | 0 | 950.51 | 72373.74 | 249890.26 | 202.28 |
| GLEGSLSADEAK | 0 | 1175.57 | 21383.14 | 24854.34 | 220.82 |
| GLEGSLSADEAKEVEK | 1 | 1660.82 | 92481.90 | 112532.09 | 174.59 |
| GLEGSLSADEAKEVEKYQK | 2 | 2080.03 | 7733.73 | 25737.37 | 109.42 |
| LLEDAAGGEFK | 0 | 1148.57 | 118569.80 | 116845.21 | 185.60 |
| LLEDAAGGEFKVFK | 1 | 1522.80 | 1015.00 | 6385.56 | 90.73 |
| QVISTAASLTGK | 0 | 1174.66 | 104828.89 | 108591.97 | 172.10 |
| QYSPASMRPIK | 1 | 1276.66 | 46278.74 | 32027.58 | 125.74 |
| VAGLEPQQVTDLVVAFHK | 0 | 1950.06 | 50993.91 | 107487.87 | 206.80 |
| VFKEGDDVDGVHWYGHHTQAPTGYSLYVFR | 1 | 3479.63 | 0.00 | 8891.45 | 57.69 |
| YEHERGDLFVALAR | 1 | 1674.85 | 20117.39 | 55067.46 | 109.84 |
|  |  |  | 798930.1 | 1273361.7 |  |
